# Exploring the Prevalence of Antimicrobial Resistance in the Environment Through Bonelli’s Eagles (*Aquila fasciata*) as Sentinels

**DOI:** 10.3390/antibiotics14080734

**Published:** 2025-07-22

**Authors:** Barbara Martin-Maldonado, Ana Marco-Fuertes, Laura Montoro-Dasi, Laura Lorenzo-Rebenaque, Jose Sansano-Maestre, Jaume Jordá, Daniel Martín Solance, Fernando Esperón, Clara Marin

**Affiliations:** 1 Department of Veterinary Medicine, School of Biomedical and Health Sciences, Universidad Europea de Madrid, 28670 Villaviciosa de Odón, Spain; barbara.martin-maldonado@universidadeuropea.es (B.M.-M.);; 2 Facultad de Veterinaria, Instituto de Ciencias Biomédicas, Universidad Cardenal Herrera-CEU, CEU Universities, 46113 Alfara del Patriarca, Spain; ana.marcofuertes@uchceu.es (A.M.-F.); laura.montoro@uchceu.es (L.M.-D.); jaume.jorda@uchceu.es (J.J.); 3 Institute of Science and Animal Technology, Universitat Politècnica de Valencia, 46022 Valencia, Spain; 4 Department of Animal Production and Public Health, Faculty of Veterinary Medicine and Experimental Sciences, Universidad Católica de Valencia San Vicente Mártir, 46018 Valencia, Spain

**Keywords:** One Health, *E. coli*, zoonoses, wildlife, multidrug resistance

## Abstract

**Background/Objectives:** Increasing levels of antimicrobial resistance (AMR) have recently been observed at the human–domestic animal–wildlife interface. Wild birds have been identified as carriers of antimicrobial-resistant bacteria and serve as excellent biomarkers for epidemiological studies. This study assessed the current AMR presence in Eastern Spain’s commensal *Escherichia coli* isolated from free-ranging Bonelli’s eagles (*Aquila fasciata*). **Methods**: Nestlings and their nests were intensively sampled between 2022 and 2024 to determine their AMR profile and characterize *E. coli*. AMR testing was conducted using the broth microdilution method, following the European Committee on Antimicrobial Susceptibility Testing guidelines. Additionally, the presence of *eae*A (intimin gene) and *stx*-1 and *stx*-2 (shiga toxins) was analyzed by real-time PCR to classify *E. coli* strains into enteropathogenic (EPEC) and Shiga-toxigenic (STEC) pathotypes. **Results**: Of all *E. coli* isolates, 41.7% were resistant to at least one antimicrobial, and 30% were multidrug-resistant. Only two strains were classified as EPEC and none as STEC. The highest resistance rates were observed for amoxicillin and tetracycline (19.6% each). Alarmingly, resistance to colistin and meropenem, last-resort antibiotics in human medicine, was also detected. **Conclusions**: Although the mechanisms of resistance acquisition remain unclear, transmission is likely to occur through the food chain, with synanthropic prey acting as intermediary vectors. These results highlight the role of Bonelli’s eagles as essential sentinels of environmental AMR dissemination, even in remote ecosystems. Strengthening One Health-based surveillance is necessary to address AMR’s ecological and public health risks in wildlife.

## 1. Introduction

Nowadays, antimicrobial resistance (AMR) is considered the most significant challenge to modern medicine, primarily due to the widespread misuse and overuse of antibiotics over the past century in hospitals, livestock, and agriculture [1]. The most critical issue is the failure to treat infections, but it has been demonstrated that AMR can also induce gut microbiota modifications that lead to gastrointestinal disorders [2,3]. Consequently, the spread of antimicrobial-resistant bacteria (ARB) poses an essential threat to human health. Nearly five million human deaths were indirectly attributed to AMR, with varied distribution around the world. While the AMR rate in Australia is 28.0 per 100,000 inhabitants, in western sub-Saharan Africa, this rate increases to 114.8 [4,5]. Similarly, the highest levels of AMR in animals are found in low- and middle-income countries, such as India and China [6]. Clinical and subclinical, unsuccessfully treated infections in livestock mean a loss of productivity and social consequences [3]. In this sense, predictions reported that the cost of AMR issues could reach up to USD 1 trillion annually worldwide by 2050, including only hospital bills, and more than USD 100 trillion considering the productivity losses [7]. 

For that reason, monitoring programs and regulatory legislation have been implemented in many countries [8]. *Escherichia coli* (*E. coli*) is one of the most employed sentinel bacteria, and it is a common microbiota species in most homeothermic species. Nevertheless, in some circumstances, some genotypes could be pathogenic for humans and animals and even zoonotic, causing infections in the urinary tract, respiratory system, gastrointestinal tract, and, in some cases, septicemia [3]. As a result, *E. coli* is one of the most common ARB isolated from lesions in humans and animals and one of the six main lethal resistant bacteria in humans [5,8,9]. 

The emergence of ARB has been frequently correlated with anthropogenic activities such as agriculture, livestock, urbanization, or mismanagement of medical residues [10,11]. Antibiotic residues can easily contaminate soils and aquatic environments from manure and wastewaters of farms, slaughterhouses, and even landfills, entering the water cycle and reaching clouds through which they may disseminate to other regions or continents [12,13,14,15]. In particular, scavenging birds such as gulls and vultures often forage at landfills, where they may be exposed to these residues and contribute to the dissemination of antimicrobial-resistant bacteria [16,17]. Furthermore, urban wildlife like pigeons or white storks has closer contact with human activities and can act as a bridge species between cities and ecosystems, acquiring ARB and spreading to other wildlife species [18]. In this context, the spread of ARB across the ecosystems is possible and easy, especially when the anthropization level is higher [10,19,20]. It remains unclear whether the presence of AMR in wildlife adversely affects biodiversity and, in turn, conservation efforts, and the role of wild animals in the dissemination and maintenance of AMR remains poorly understood [21]. For this reason, studies assessing the AMR present in wildlife species, especially in those considered sentinels of the environment, are key in the surveillance of AMR under the One Health approach. 

Wild birds are considered useful bioindicators of ecosystem health. Among them, birds of prey are good sentinels for such studies due to their position at the top of the trophic chain and the large territory they usually cover [22]. Specifically, Bonelli’s eagle (*Aquila fasciata*) usually nests in rocky areas and cliffs in low-human-density regions far from urban centers. However, it makes daily movements across territories that average up to 80 km^2^ [23,24]. Its diet is based on rabbits, partridges, and pigeons, which are more linked to human activities. Still, it can also feed on small insectivorous and omnivorous birds, so it can be considered a super-predator [22]. Furthermore, it is a threatened species listed as Least Concern in the IUCN Red List with a decreasing trend of the wild populations due to pesticides, prey decline, habitat disturbances, and hunting [25]. Thus, from a One Health standpoint, Bonelli’s eagles could be considered an effective bioindicator of AMR in the environment, owing to their extensive mobility and minimal exposure to antibiotics [18]. 

For these reasons, this study aimed to assess the current epidemiological AMR situation in commensal *E. coli* isolated from free-ranging Bonelli’s eagles in Eastern Spain. 

## 2. Results

**Figure 1:**
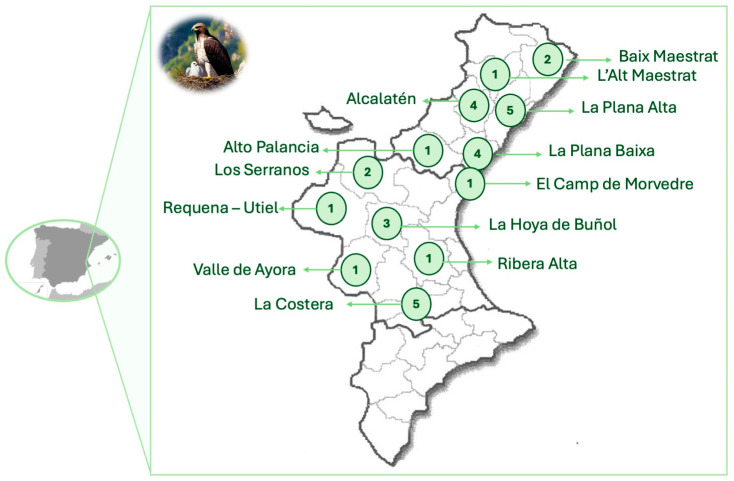
Spatial distribution of Bonelli’s eagle (*Aquila fasciata*) nests across the districts in the Valencia and Castellón provinces.

Overall, 31 nests and 50 nestlings were examined in this study. According to the sampling year, 6 nests and 11 nestlings were sampled in 2022, 14 nests and 20 nestlings in 2023, and 11 nests and 19 nestlings in 2024. According to the region, 18 nests were from Castellón, and 14 were from Valencia. Figure 1 shows that Bonelli’s eagle nests are pretty well distributed throughout the territory of both provinces, Valencia and Castellón, indicating a widespread occupation of available habitats within the study area. Of the 31 nests, fresh feces could be collected from 27 nests (Supplementary Table S1).

*E. coli* was recovered from all the nests (27/27) and 48 nestlings (48/50); both negative nestlings were born in 2023. Of the 75 *E. coli* strains isolated, only 2 were identified as zoonotic enteropathogenic *E. coli* (EPEC), and none of the *E. coli* isolates were zoonotic Shiga-toxigenic *E. coli* (STEC). One of the EPEC strains was considered multidrug-resistant (MDR), while the other was susceptible to all antimicrobials (Supplementary Table S1).

### 2.1. Global Antimicrobial Resistance Results

**Figure 2:**
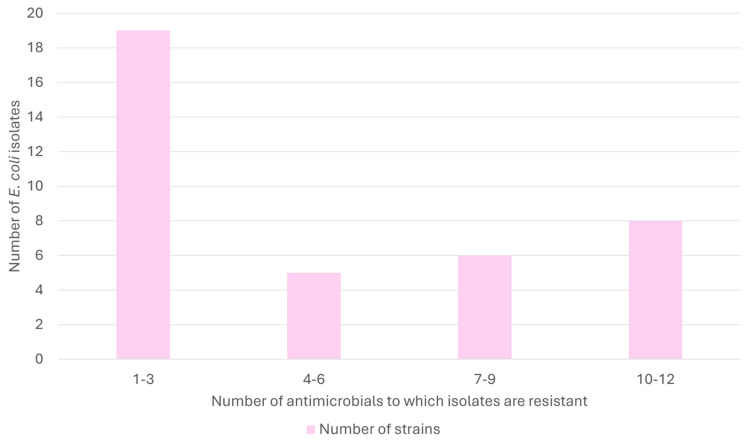
Resistance profiles of *E. coli* isolated from Bonelli’s eagles (*Aquila fasciata*): strain counts by antimicrobial range.

Overall, 53.3% (40/75, CI_95%_ 42.2–64.2%) of the isolates were resistant to at least one antimicrobial. All the antimicrobials included in the analysis showed some level of resistance. MDR was observed in 29.3% of the isolates (22/75, CI_95%_ 20.2–40.4%). Three isolates were resistant to 12 antimicrobials, and one included 10 antimicrobial classes (only macrolides and carbapenems were effective) (Figure 2).

**Figure 3:**
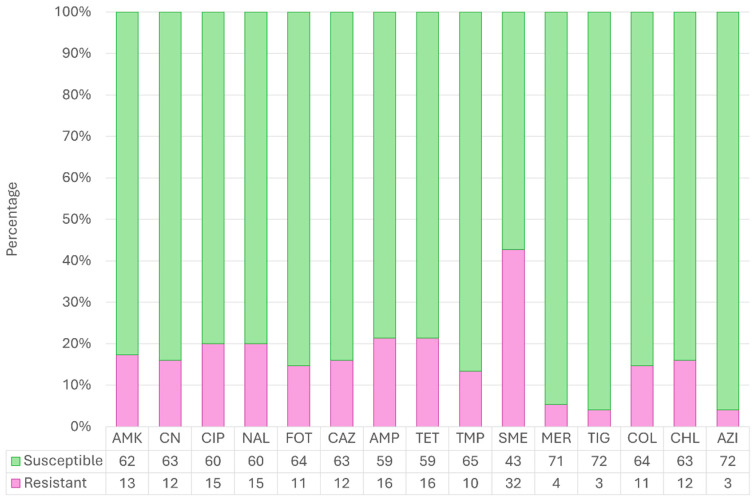
Details of the antimicrobial resistance proportions to the 15 antimicrobials among the 76 *E. coli* isolates from Bonelli’s eagles (*Aquila fasciata*) (2022–2024). AMK: amikacin; CN: gentamicin; CIP: ciprofloxacin; NAL: nalidixic acid; FOT: cefotaxime; CAZ: ceftazidime; AMP: ampicillin; TET: tetracycline; TMP: trimethoprim; SME: sulfamethoxazole; MER: meropenem; TIG: tigecycline; COL: colistin; CHL: chloramphenicol; AZI: azithromycin.

As has been represented in Figure 3, the higher AMR rate was observed for sulfamethoxazole (42.67%, 32/75, CI_95%_ 32.1–53.9%), followed by ciprofloxacin, nalidixic acid, and tetracycline (20%,15/75, CI_95%_ 12.5–30.4% each one), amikacin (17.33%, 13/75, CI_95%_ 10.4–27.43%), and chloramphenicol, ceftazidime, and gentamycin (16%, 12/75, CI_95%_ 9.4–25.9% each one). In contrast, tigecycline, azithromycin, and meropenem were the antimicrobials with the lowest resistance.

**Figure 4:**
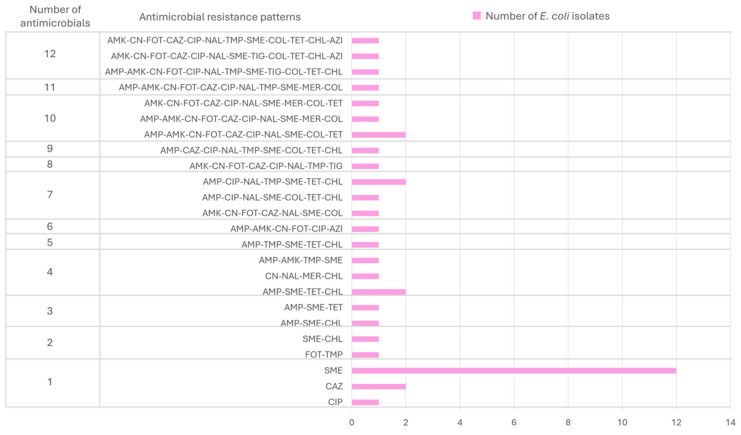
Antimicrobial resistance patterns among the *E. coli* strains isolated from Bonelli’s eagles (*Aquila fasciata*). AMK: amikacin; CN: gentamicin; CIP: ciprofloxacin; NAL: nalidixic acid; FOT: cefotaxime; CAZ: ceftazidime; AMP: ampicillin; TET: tetracycline; TMP: trimethoprim; SME: sulfamethoxazole; MER: meropenem; TIG: tigecycline; COL: colistin; CHL: chloramphenicol; AZI: azithromycin.

The diversity of MDR patterns was high, with 19 different combinations, and only 1 of them was observed twice (AMK-CN-AMP-FOT-CAZ-CIP-NAL-SME-COL-TET) (Figure 4, Supplementary Table S1). These isolates with the same pattern belonged to nestlings from different geographical areas. The EPEC MDR strain was resistant to 12 antimicrobials from 10 different classes, and its pattern was AMK-CN-AMP-FOT-CIP-NAL-TMP-SME-TIG-COL-TET-CHL.

Among the variables assessed, the presence of AMR was not associated with the region where the samples were collected (*p* = 0.305). Regarding the year of sample collection, no statistical differences were observed between the presence of AMR or MDR and the different years (*p* = 0.335; *p* = 0.281, respectively).

### 2.2. Antimicrobial Resistance Fluctuation Through the Years

**Figure 5:**
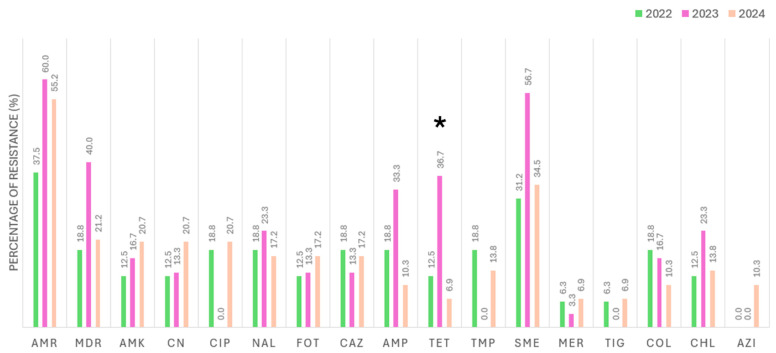
Percentage of resistance detected among the *E. coli* isolates obtained from Bonelli’s eagles (*Aquila fasciata*) from 2022, 2023, and 2024 according to each antimicrobial. AMR: antimicrobial resistance; MDR: multidrug resistance; AMK: amikacin; CN: gentamicin; CIP: ciprofloxacin; NAL: nalidixic acid; FOT: cefotaxime; CAZ: ceftazidime; AMP: ampicillin; TET: tetracycline; TMP: trimethoprim; SME: sulfamethoxazole; MER: meropenem; TIG: tigecycline; COL: colistin; CHL: chloramphenicol; AZI: azithromycin. The symbol * highlights the antimicrobials with statistical differences between years.

Of the 20 nests sampled between 2022 and 2024, 13 were sampled in 2022, 5 in 2023 and 2024, and 2 were sampled in all three years. Overall, no statistically significant fluctuations were observed in the presence of AMR across the years (*p* = 0.335). Nevertheless, the highest rates were observed in 2023 when assessing the AMR to each antimicrobial, specifically for tetracycline (*p* = 0.004). Also, it is noteworthy that resistance to azithromycin was detected only in 2024. Details about the AMR and MDR found each year are summarized in Figure 5. 

## 3. Discussion

From a One Health perspective, wild birds have been proposed as good bioindicators of AMR in the ecosystem due to their wide-ranging movements and low antibiotic exposure [18]. Nowadays, the scientific literature about AMR on wild birds is scarce, even more so on threatened species. Most of the research has been focused on Passeriformes, Charadriiformes, and Anseriformes orders, as they are considered migratory or urban birds and thus good sentinels [18,26,27]. In contrast, a few included birds of prey, which make excellent daily dispersion through their territory, and are at the top of the trophic pyramid [20,28,29,30]. Previous studies on Bonelli’s eagles reported AMR but linked it to different zoonotic pathogens. In this sense, more than half of the *E. coli* isolates (53.3%) from the present study were resistant to at least one antimicrobial, a percentage higher than that reported for *Salmonella* in 2020 (36.8%, 7/19), but lower than for *Campylobacter* in 2021 (66,7%, 6/9) [31,32].

Approximately 35% of the isolated strains exhibited resistance to at least seven antimicrobials, indicating a bimodal distribution: most strains showed either low (1–3) or high (≥7) resistance levels, with few displaying intermediate resistance. This pattern suggests a polarized resistance profile, potentially reflecting the population’s distinct ecological or evolutionary pressures. Ecological studies have documented bimodal distributions, often arising from heterogeneous environments or varying selective pressures [33]. Interestingly, in 2023, the proportion of resistant *E. coli* isolates was slightly higher but statistically insignificant (Figure 5). This increase could be related to annual variations in prey availability, urban pigeon populations near nesting areas, or changing environmental contamination levels. The small sample size could also amplify inter-annual differences. Therefore, while this finding is noteworthy, further monitoring is needed to determine whether it represents an isolated fluctuation or an emerging trend. This aligns conceptually with the Pareto principle, where a minority of individuals harbor most resistance, potentially indicating that resistance is endemic within this wild Bonelli’s eagle population [34]. For instance, although our study revealed a general stability in the resistance profiles over time, the higher proportion observed in 2023 suggests that annual ecological or environmental factors might still influence these patterns. This may imply that resistance is well-established within the wild Bonelli’s eagle population, possibly reflecting endemic characteristics. 

Moreover, 19 different AMR patterns were described. MDR (29.3%) was in concordance with that reported in other birds of prey species [30]. The EPEC MDR strain was also resistant to 12 antimicrobials from 10 different classes. The gene *eae*A is located on a chromosomal pathogenicity island (LEE, locus of enterocyte effacement), whereas most antimicrobial resistance genes are found on plasmids. Although some studies have reported no clear association between *eae*A and resistance genes, others have observed co-occurrence, especially in strains exposed to selective pressures [35]. This may reflect ecological co-selection, where selective pressures favor strains harboring both virulence and resistance traits, even when these genes are located on separate genetic elements. Our findings contribute to this body of evidence and raise questions about the mechanisms driving co-selection or potential genetic linkage between resistance determinants and virulence factors in *E. coli*.

Overall, resistance to all the antimicrobials included in the antimicrobial susceptibility test panel was recorded, including those categorized by the World Health Organization as highly important, critically important, and with the highest priority critically important [36]. Resistance was detected for tigecycline and meropenem, two last-resort antimicrobials in human medicine [36]. These results are very concerning considering the low contact Bonelli’s eagle has with humans or domestic animals. The exposition of wild raptors to antimicrobials has been proposed through their prey, which could be more synanthropic and can acquire resistance from environments with high antimicrobial pressure (i.e., urban areas, landfills, farms, or wastewater treatment plants) [20,37,38]. However, in addition to the wide distribution of nests across both provinces, it is important to consider that Bonelli’s eagle territories can range from approximately 36 km^2^ (Kernel method) to 50 km^2^ (MCP method) [39]. Given that both Valencia and Castellón are provinces with high human population densities, the probability of nests being located near urban or rural settlements is relatively high, despite the species’ low level of interaction with human activities.

The antimicrobial class with the highest resistance was sulfonamides, represented by sulfamethoxazole, doubling the resistance rates of penicillins, tetracyclines, and quinolones, in opposition to most of the studies in wild birds [26,30,38,40,41]. This finding contrasts with the extensive use of penicillins, tetracyclines, and quinolones in public and animal health, and the high levels of AMR typically reported against them [8,42]. Inhabiting cities, feeding on landfills, or breeding in buildings could enhance the ARB acquisition for synanthropic birds [13,18]. In this sense, nowadays, the main prey of Bonelli’s eagles is pigeons more than partridges or rabbits [31,43]. As pigeons are synanthropic and ubiquitous species, their contact with human residues in cities or with manure and wastewater from livestock is very close, playing a key role as a bridge species between human activities and wildlife [31,37]. 

Resistance to sulfamethoxazole was much higher than most previous reports in wild birds from other countries [26,30,44]. However, a few studies found high proportions of trimethoprim/sulfamethoxazole resistance in raptors from Spain (41.4%), Greece (91.7%), and Italy (83.7%) [29,40,41]. This situation could be linked to the colibacillosis treatment with sulfamethoxazole in humans and animals [3]. Also noteworthy, the resistance to azithromycin was recorded only in 2024, when human consumption has been stable over the last ten years. Azithromycin is not a common antimicrobial in veterinary practice, and azithromycin resistance rates are low, including wild birds [8,38].

Despite the low and moderate rates, it is concerning that resistance was observed to meropenem and colistin, respectively. Those are two key antimicrobials for treating human multidrug-resistant infections [36]. Resistance to meropenem in animals, including wild birds, is rare, and the acquisition pathways are unclear as this antimicrobial is restricted only for human treatment [8,29,36,40,45]. In contrast, several studies have reported colistin resistance in wild birds of prey, such as griffon vultures (*Gyps fulvus*) or black kites (*Milvus migrans*), and synanthropic birds, such as white storks (*Ciconia ciconia*) and pigeons [18,46,47]. Despite its actual ban in many countries, colistin has been employed as a growth promoter in livestock for decades, fostering the development of resistance against it, which has been evidenced by the presence of the *mcr*-1 gene in *E. coli* from pig carcasses [48]. As vertical transmission of AMR genes is possible, the origin of these colistin-resistant isolates could stem from the past [20]. However, the last European reports about AMR noticed very low colistin resistance in livestock, including poultry, cattle, and pigs [8]. In contrast, its administration to humans in hospitals has doubled in the last ten years due to the increase in MDR infections, so the environmental pressure may not be as reduced as previously thought [42]. 

Finally, this study presents some limitations inherent in research on wildlife. Firstly, the number of animals analyzed is limited due to the free-living population in Eastern Spain. However, all the monitored nests were included in this research. Additionally, analyses based on bacterial culture usually introduce bias by selecting just one or a few single colonies to work with, ignoring other strains of the same bacterial species. These limitations highlight the need for more integrated and well-funded strategies in AMR surveillance programs for a deeper and broader analysis of AMR in wildlife populations. 

## 4. Materials and Methods

### 4.1. Study Population and Sampling Collection

During breeding seasons of 2022, 2023, and 2024, Bonelli’s eagle nests from Eastern Spain (Castellón and Valencia) were inspected as part of the Protocol for Marking and Disease Analysis in Bonelli’s Eagle Nestlings, an official species conservation program led by the regional government. This monitoring was conducted by the High-Altitude Intervention Group, a team of official forest rangers and climbers, due to the species’ tendency to nest on high cliffs. This governmental conservation program includes routine nest inspections, clinical examinations, banding, and sampling for pathogen monitoring, without any additional handling of the animals beyond standard conservation procedures. The present study results from a parallel study using the same samples. Thus, ethical approval was not mandatory. 

During the clinical examination, a cloacal sample was obtained from each nestling. To this end, a sterile cotton swab was introduced 1 cm into the cloaca and slowly rotated to obtain the maximum sample possible. All handling procedures followed the animal welfare guidelines outlined in European Directive 2010/63/EU [49]. Then, the swab was preserved in Aimies medium. Additionally, a fresh fecal sample was collected from each nest and stored in a sterile container when available. All samples were kept at 4 °C and analyzed in the laboratory within 24 h of collection. Data on the number of nestlings, locations, and observed incidents were systematically recorded. 

### 4.2. Escherichia coli Isolation and Characterization 

*E. coli* isolation was performed within 48 h after the sample collection. Swabs were first pre-enriched in buffer peptone water (BPW, Scharlau^®^, Barcelona, Spain) for 24 h at 37 ± 1 °C, and then streaked into tryptone bile X-glucuronide agar (TBX; Scharlau^®^, Barcelona, Spain). Fresh feces were first homogenized and weighed, and BPW was added at a ratio of 1:10 *v*/*v* for pre-enrichment for 24 h at 37 ± 1 °C. As with the swabs, the pre-enriched broth was transferred to TBX. All the plates were incubated at 37 ± 1 °C for 24 h. A single suspected colony (rounded blue-green brilliant) was collected from each plate and streaked into a nutritive agar (Scharlau^®^, Barcelona, Spain) to obtain a monoclonal culture. After 24 h of incubation at 37 ± 1 °C, presumptive *E. coli* isolates were identified using the API 20E system (bioMérieux, Marcy-l’Étoile, France) according to the manufacturer’s instructions. Biochemical profiles were analyzed with the APIweb^®^ software (version 5.0), and only isolates with an identification probability of ≥95% as *E. coli* were included for further analysis. 

Then, for molecular characterization of *E. coli* strains, DNA extraction was performed according to Dashti et al. [50]. Briefly, 5–10 colonies of overnight growth *E. coli* were dissolved into 1.5 mL of phosphate-buffered saline (PBS) solution (Invitrogen, Thermo Fisher Scientific^®^, Madrid, Spain) and centrifuged at 1000 rpm for 5 min. The supernatant was conserved for the following analysis. Real-time PCRs based on TaqManTM probes were performed to identify zoonotic EPEC and STEC *E. coli*. The partial amplifications of the *eae*A, *stx*-1, and *stx*-2 genes were performed according to the information summarized in Table 1. Sterile deionized water was used as a negative control, and an internal positive control—obtained from a bird sample previously tested and sequenced carrying the three target genes—was included in each run. All PCR protocols employed probe-based assays to ensure specific amplification and accurate positive and negative results interpretation. If the sample was positive for *eae*A and one of the *stx* genes, the strain was considered STEC, but if the sample was positive only for *eae*A, the strain was considered EPEC. 

### 4.3. Antimicrobial Susceptibility Test

According to the European Committee on Antimicrobial Susceptibility Testing (EUCAST) guidelines, antimicrobial susceptibility tests were performed using the broth microdilution method. Each *E. coli* strain was previously cultured in nutritive agar (Scharlau^®^, Barcelona, Spain) at 37 ± 1 °C for 24 h and subjected to Sensititre EUVSEC3^®^ plates (ThermoFisher Scientific^®^, Madrid, Spain) following the manufacturer’s instructions. A total of 15 antimicrobials from 12 different classes were tested: 2 aminoglycosides (amikacin and gentamicin), 2 quinolones (ciprofloxacin and nalidixic acid), 2 cephalosporines (cefotaxime and ceftazidime), 1 penicillin, 1 tetracycline, 1 pyrimidine, 1 sulfonamide, 1 carbapenem, 1 glycylglycine, 1 polymyxin, 1 amphenicol, and 1 macrolide (Table 2). The lowest concentration of the agent that completely inhibits visible growth was considered the minimum inhibitory concentration (MIC) for each strain and compared to the cut-off values of the last version of EUCAST guidelines to classify them as resistant or sensitive to each antimicrobial [52]. For the antimicrobials without cut-off values in EUCAST tables (nalidixic acid and tetracycline), the breakpoint was established following the Clinical and Laboratory Standards Institute (CLSI) guidelines [53].

Resistance was considered when a strain was non-susceptible to one antimicrobial and MDR exhibited resistance to at least one antimicrobial agent from three or more antimicrobial classes [8].

### 4.4. Statistical Analysis

A Generalized Linear Model (GLM) was performed to assess the influences on the detected AMR patterns. Assuming a binomial distribution, the probit link function was used to determine whether there was an association with the categorical variables (year of sampling and location of the nest). A *p*-value ≤ 0.05 was considered to indicate a statistically significant difference. Results are expressed as absolute (n) and relative (%) frequencies, including 95% confidence intervals (CI_95%_) for each one. All the statistical analyses were performed using the SPSS 25.0 software package (SPSS Inc., Chicago, IL, USA, 2002).

## 5. Conclusions

The findings of this study reveal the concerning presence of AMR in *E. coli* isolated from Bonelli’s eagles (*Aquila fasciata*), a species with minimal direct contact with human activities. The high AMR and MDR rates observed and the variability in AMR patterns highlight the widespread dissemination of AMR in the environment. Furthermore, resistance to colistin and meropenem, last-resort antibiotics in human medicine, is particularly alarming. While the exact mechanism of resistance acquisition remains unclear, it is likely occurring through the food chain, with synanthropic prey acting as intermediary vectors. This reinforces the need to consider not only urban or migratory species as reservoirs of resistant bacteria but also top predators that may serve as key sentinels of environmental AMR pollution. In conclusion, the need for more comprehensive environmental surveillance strategies within the One Health framework is urgent, as AMR in wildlife poses a risk to human and animal health and may still have unknown consequences for biodiversity and ecosystem balance.

## Figures and Tables

**Table 1:** Primer sequences employed in real-time PCR analysis for *E. coli* characterization [51].

Gene	Primers/Probe
*eae*A	EAE-S For 5′-ACTGGACTTCTTATTRCCGTTCTATG EAE-B1 5′-CTAAGCGGGTATTGTTACCAGA-3′ EAE- 5′-6-Fam-AATCCTGATCAATGAAGACGTTATAGCCCA-BHQ-1-3′
*stx*-1	SLT1-1 5′-CTTCCATCTGCCGGACACATA-3′ SLT1-2 5′-ATTAATACTGAATTGTCATCATCATGC-3′ SLT1-S 5′-6-Fam-AAGGAAACTCATCAGATGCCATTCTGGCA-BHQ-1-3′
*stx*-2	SLT2-1 5′-GACGTGGACCTCACTCTGAACTG-3′ SLT2-2 5′-TCCCCACTCTGACACCATCC-3′ SLT2-S 5′-6-Fam-TACTCCGGAAGCACATTGCTGATTCGC-BHQ-1-3′

**Table 2:** Antimicrobials included in the EUSVEC3^®^ plates and cut-off values (μg/L) employed for the interpretation of the results [49].

Antimicrobial Class	Antimicrobial Molecule	Abbreviation	EMA Category *	Cut-off Values (μg/mL) **
Aminoglycosides	Amikacin	AMK	C	>8
	Gentamicin	CN	C	>2
Quinolones	Ciprofloxacin	CIP	B	>0.5
	Nalidixic acid	NAL	B	>16
Cephalosporines	Cefotaxime	FOT	B	>4
	Ceftazidime	CAZ	B	>2
Penicillin	Ampicillin	AMP	D	>8
Tetracycline	Tetracycline	TET	D	>8
Pyrimidine	Trimethoprim	TMP	D	>4
Sulfonamide	Sulfamethoxazole	SME	D	>64
Carbapenem	Meropenem	MER	A	>8
Glycylglycine	Tigecycline	TIG	A	>0.5
Polymyxin	Colistin	COL	B	>2
Amphenicol	Chloramphenicol	CHL	C	>16
Macrolide	Azithromycin	AZI	C	>16

* EMA: European Medicines Agency. This column indicates the EMA categorization of antimicrobials and animal use restrictions based on European AMR reports and public health importance. Categories are A. Antimicrobials avoided for veterinary use except in some cases in companion animals; B. Antimicrobials critically important for humans with some restrictions on animals; C. Antimicrobials to be considered with caution for animal treatments; and D. Antimicrobials recommended as first use for animals. ** Strains were considered resistant when the MIC exceeded the cut-off value.

## Data Availability

The data supporting this study’s findings are available upon request from the corresponding authors.

## Supplementary Materials

The following supporting information can be downloaded at: https://www.mdpi.com/article/10.3390/antibiotics14080734/s1. Supplementary Table S1. Individual data for all Bonelli’s eagles (*Aquila fasciata*) included in the study, including results of *Escherichia coli* detection, strain characterization, and antimicrobial susceptibility profiles.

## Abbreviations

The following abbreviations are used in this manuscript:

AMR	Antimicrobial resistance
ARB	Antimicrobial-resistant bacteria
BPW	Buffered peptone water
EUCAST	European Committee on Antimicrobial Susceptibility Testing
EPEC	Enteropathogenic *E. coli*
GLM	General Linear Model
MDR	Multidrug-resistant
MIC	Minimum inhibitory concentration
IUCN	International Union for Conservation of Nature
STEC	Shiga-toxigenic *E. coli*
TBX	X-glucuronide agar
